# New Operation in Cases of Effusion into the Cavity of the Chest

**Published:** 1850-11

**Authors:** 


*New Operation in Cases of Effusion into the Cavity of the Chest.* By H. J. Bowditch. Communicated at a Meeting of the Boston Society for Medical Improvement.

Dr. Bowditch regretted that the lateness of the hour would prevent him from giving a detailed account of some cases that he had seen, where very excellent results had followed upon the puncture of the chest in which large effusions existed. He would, however, show an instrument which he had made after the model of one used by Dr. Morrill Wyman, of Cambridge. It consisted simply of a brass suction-pump, arranged without valves, after the stop-cock fashion. There were two apertures, one for suction and the other for a discharge pipe, and by turning the piston-pipe ninety degrees, one or the other of these apertures was opened. An exploring canula was arranged so as to fit tightly into the suction aperture; and having been introduced into the chest, the suction could be applied immediately, and all the fluid usually discharged, without any change of the apparatus, save the turning of the piston-pipe, above described, in order to discharge the fluid when the barrel was full.

Dr. B. had seen the operation performed five times during the last three months. All the patients were immediately more or less relieved; two had tubercles at the time, one of whom has since died. Two cases were perfectly successful, the patients being, at the time of the operation, very ill, with pulse over 120, night sweats, &c. One recently operated on with the greatest relief was still under treatment. Finally, he knew of one case treated by Dr. Wyman, who is now well, and who undoubtedly would have died had the operation not been performed.

The pain of this operation is, comparatively speaking, nothing, and the patients are generally not at all troubled by it. The wound, of course, closes instantly on the removal of the canula, and no air can enter the chest while the apparatus is in operation.

Dr. Bowditch concluded by remarking that operations had been done from time immemorial, upon the chest in cases of effusion, but he believed that they were generally considered as a last resort. In Guy's Hospital Reports, cases are given of the use of the trocar, but Dr. B. believed that to Dr. Wyman was due the credit of having first proposed the use of the *exploring canula with suction applied thereto.*

Dr. Bowditch has arrived at the following conclusions:—

1st. The operation is perfectly simple, but slightly painful, and can be done with ease upon any patient in however advanced a stage of disease.

2d. It should be performed forthwith in *all* cases in which there is a complete filling up of one side of the chest.

3d. Dr. B. had determined to use it in *any* case of even *moderate* effusion lasting more than a few weeks, and in which there should seem to be an indisposition to yield to the ordinary modes of treatment.

4th. Dr. B. would urge the practice of puncturing in this way upon the medical profession *as a very important measure in practical medicine;* for he believed that, by this method, death may be frequently prevented from ensuing either from sudden attacks of dyspnœa or subsequent phthisis, or, finally, as in two cases he had seen, from the gradual wearing out of the powers of life from inability to absorb the fluid, even when all the organs, save the pleura of the affected side, were healthy.

5th. Dr. B. was likewise inclined to the belief that this operation if generally adopted, would sometimes prevent the occurrence of those very tedious cases of spontaneous evacuation of purulent fluid, and those great contractions of the chest that occur after long-continued effusion and the subsequent discharge or absorption of a fluid.—*Am. Jour. of Med. Sciences.*

